# Critical Considerations for Investigating MicroRNAs during Tumorigenesis: A Case Study in Conceptual and Contextual Nuances of miR-211-5p in Melanoma

**DOI:** 10.3390/epigenomes7020009

**Published:** 2023-04-26

**Authors:** Fatemeh Vand-Rajabpour, Meghan Savage, Rachel L. Belote, Robert L. Judson-Torres

**Affiliations:** 1 Department of Medical Genetics, School of Medicine, Tehran University of Medical Sciences, P.O. Box 14155-6447, Tehran 14176-13151, Iran; 2 Department of Oncological Sciences, University of Utah, Salt Lake City, UT 84112, USA; 3 Huntsman Cancer Institute, University of Utah, Salt Lake City, UT 84112, USA; 4 Department of Dermatology, University of Utah, Salt Lake City, UT 84112, USA

**Keywords:** microRNA, melanoma, cancer progression, miR-211, melanocytes

## Abstract

MicroRNAs are non-coding RNAs fundamental to metazoan development and disease. Although the aberrant regulation of microRNAs during mammalian tumorigenesis is well established, investigations into the contributions of individual microRNAs are wrought with conflicting observations. The underlying cause of these inconsistencies is often attributed to context-specific functions of microRNAs. We propose that consideration of both context-specific factors, as well as underappreciated fundamental concepts of microRNA biology, will permit a more harmonious interpretation of ostensibly diverging data. We discuss the theory that the biological function of microRNAs is to confer robustness to specific cell states. Through this lens, we then consider the role of miR-211-5p in melanoma progression. Using literature review and meta-analyses, we demonstrate how a deep understating of domain-specific contexts is critical for moving toward a concordant understanding of miR-211-5p and other microRNAs in cancer biology.

## 1. Introduction

Protein-coding genes constitute less than 3% of the human genome [1,2], yet nearly 75% of the genome is transcribed as RNA [3]. In the three decades since the groundbreaking discovery that a small non-coding RNA could regulate gene expression in metazoans [4], myriad classes and functions of non-coding RNAs—the so-called “dark matter transcriptome”—have been revealed and explored [5]. MicroRNAs (miRNAs) are the most abundant of the small non-coding RNAs [3]. Their emergence and expansion in genomes are strongly correlated with the diversification of the complex body plans of bilaterians [6,7,8,9,10,11,12,13]. Both this evolutionary history and their expression patterns during embryonic development suggest miRNAs orchestrate rapid and robust transitions between closely related but distinct transcriptional states of specific cells [6,13,14,15,16,17,18,19,20]. Given that tumorigenesis is in large part comprised of an ordered reversal of developmental programs [21,22], it is therefore not surprising that, across cancers, disease progression is associated with the dysregulation of miRNAs [23,24,25,26,27].

The molecular function of miRNAs is to guide the ribonucleoprotein RNA-induced silencing complex (RISC) to specific protein-coding mRNAs, thereby dampening their translation [28]. Specificity is achieved by binding of the targeting miRNA to the targeted mRNA. This interaction requires only partial complementarity [29]. Consequently, hundreds of genes can be targeted by a single miRNA, and over 60% of human protein-coding genes are regulated by RISC [30,31]. The presence of miRNAs has applied profound evolutionary pressure on mRNAs to be either selectively maintained or selectively excluded from the network of co-regulated genes targeted by each miRNA [14,30,32,33]. In addition, predictive algorithms can determine the likely targets of a miRNA based on the mRNA sequence [29,30,34,35]. This combination of evolutionary and molecular features has inspired abundant investigations into the utility of miRNAs for detecting and classifying tumors, for pleiotropic suppression of tumor-promoting genes, and for identifying the complex gene networks that regulate tumorigenesis [23,36,37,38].

Unfortunately, despite the potential, investment, and enthusiasm, miRNA-based diagnostic, prognostic, and therapeutic breakthroughs in oncology have yet to be realized [37,39,40]. Tens of thousands of studies investigating the use of miRNAs as diagnostics have been published [38]. However, in oncology, only a single product—designed for the stratification of thyroid cancers—has emerged as a miRNA-based diagnostic both available to clinicians and covered by insurance companies [41]. A comparable number of publications describing miRNA-based therapeutic strategies have yielded only five phase I clinical trials (NCT01829971, NCT02369198, NCT02580552, NCT03884556, NCT05429073) [38,42,43,44]. In comparison, as of 2020, 57 clinical trials for therapeutics based on small interfering (si)RNA were ongoing or completed, including 16 in phase II and 8 in phase III [45]. Since siRNA and miRNA share nearly identical chemical and pharmacokinetic properties, it would appear the comparatively modest pace of miRNA-based therapeutic development and translation is not merely the consequence of the technical challenges associated with the clinical administration of RNA molecules.

There exists another more fundamental explanation for the poor adoption of miRNA-based diagnostics and therapeutics—the inconsistency of reported observations surrounding miRNA expression and function. In the field of oncology, corroboration between studies of miRNA expression is tenuous at best, with diagnostic or prognostic signatures demonstrating high rates of discordance [46,47,48]. The prevalence of contradictory observations regarding the relative oncogenic or tumor-suppressive role of specific miRNAs in cancer progression is evident from the widespread use of titular hedging descriptors in reviews that summarize the literature (examples include: “*opposing roles*”, “*paradoxical behavior*”, or “*dual-role regulator*”) [49,50,51,52,53,54,55]. In short, the production of ostensibly conflicting observations from different research groups has become the expected, not the exceptional, outcome of miRNA studies.

There are technical and analytical challenges associated with miRNA research that explain some of the discordances in reported observations. These challenges have been well-reviewed and include confusing nomenclature, distinct biases across different methods for isolating and profiling miRNA, and a lack of normalization standards, among others [40,46,48,56,57]. However, experimental hardships and nuances are certainly not unique to miRNA research. Are there other explanations for the frequency of contradictory results that are inherent to miRNA biology? Here, we review the theory that the biological function of miRNAs is to confer robustness to specific cell states. Using melanoma as a case study, we demonstrate that when interpreted through the lens of “cell state buffer” as opposed to “gene inhibitor”, ostensibly controversial miRNA literature instead harmonizes.

## 2. The Role of MicroRNAs in Stabilizing Transcriptional Programs

### 2.1. MicroRNAs Bolster Cell State Robustness

It is tempting, and indeed commonplace, to represent the relationship of a miRNA to its target as that of a ‘subtle inhibitor’—comparable in concept if not in potency to well-established negative feedback circuits (e.g., inhibition of Smoothened by Patched); targeted protein phosphorylation and/or degradation (e.g., phosphorylation of NF1 by PKC); or small molecule enzyme inhibitors (e.g., binding of tyrosine kinases by imatinib). When multiple targets of a miRNA are identified, the miRNA is frequently depicted as a pleiotropic inhibitor—similar to a promiscuous kinase inhibitor. However, when a gene is targeted by a miRNA, protein output is typically reduced by about two-fold [58]. This level of change is relatively modest, especially considering that the loss of a single functional allele of a gene usually results in a recessive trait, suggesting that cells are generally equipped to tolerate or compensate for two-fold changes in the expression of most genes. Thus, while the direct *molecular* function of a miRNA is to inhibit the expression of the target mRNAs, the *cellular* function of the miRNA—that is, the net effect of the miRNA in the regulation of genetic programs and, thereby, cell behavior—is conceptually more complex [59]. The key difference between the consideration of a molecular function and a cellular function is that of context—the ecosystem within the cell. In the case of a miRNA, the cellular ecosystem consists of, at minimum, the dynamic expression, processing, and availability of each target gene, all other miRNAs, and the protein components of RISC. Both theoretical and experimental evidence has demonstrated that when these higher-order interactions are considered, the cellular role of a miRNA is more accurately described as bolstering cell state robustness as opposed to inhibiting gene expression [59,60,61].

**Figure 1:**
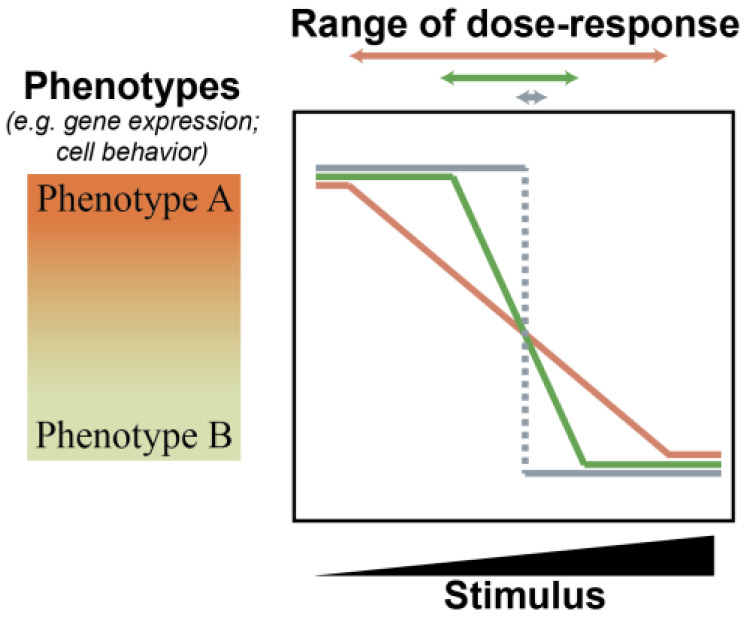
Thresholds in cellular responses to a stimulus. Three theoretical models describing a cellular response to a stimulus are depicted. For each, a cell can present variable degrees of Phenotype A (for example, expression of a gene or a particular behavior) or Phenotype B, dependent on the dose of a stimulus. In a response without thresholding (orange line), incremental changes in the stimulus result in incremental shifts in the full phenotype spectrum. As the threshold effect increases (from orange to green to grey), the range in which incremental changes in stimulus dose result in observed changes in phenotype narrows (corresponding double headed arrows).

The robustness of a cell state refers to the ability to maintain a specific phenotype despite internal or external perturbations that affect gene expression. However, being robust does not mean being static. Living organisms are dynamic, and cells and tissues have evolved to respond to particular perturbations with orchestrated changes. Therefore, measuring cell state robustness should be considered as a threshold, below which perturbations do not cause a change in cell state and above which perturbations cause a change in behavior (Figure 1). For example, a robust cell will not deviate from its healthy and routine daily behaviors even if it receives an aberrant growth factor signal or acquires a genetic amplification of an oncogene. In contrast, a cell with a low degree of robustness might change its behavior as a consequence of a bout of transcriptional noise.

**Figure 2:**
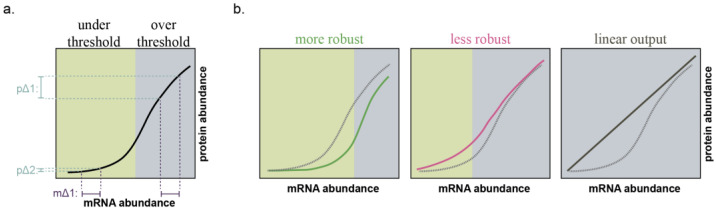
MicroRNAs introduce a threshold effect to protein translation. (**a**) mRNAs targeted by miRNAs possess a “robustness threshold”. If mRNA abundance is under the threshold (green background), protein translation is non-linear. Here, a change in mRNA abundance (mΔ1) results in a miniscule change in protein translation (pΔ2). If mRNA abundance is over the threshold (grey background), protein translation is linear, such that the same change in mRNA abundance (mΔ1) results in a comparable change in protein translation (pΔ1). (**b**) An increase in miRNA functional units increases robustness (green line); a decrease in miRNA functional units decreases robustness (red line); and for genes lacking miRNA regulation, protein translation is not buffered against fluctuations in mRNA abundance (black line). Dotted line indicates curve from A. Informed by Ebert and Sharp (2012) [59] and Mukherji, et al. (2011) [61].

MicroRNAs provide a molecular mechanism to establish a common threshold across a network of genes [59,60,61]. For each individual gene, if targeting miRNAs are present, a change in mRNA abundance does not necessarily lead to changes in protein abundance, thereby providing stability to the system [59,61]. However, if the mRNA levels increase to such an extent that the miRNAs present are no longer sufficient to saturate all target binding sites, the system “toggles”. This results in a considerable increase in protein levels and subsequent translation that is proportional to the mRNA molecules present (Figure 2).

**Figure 3:**
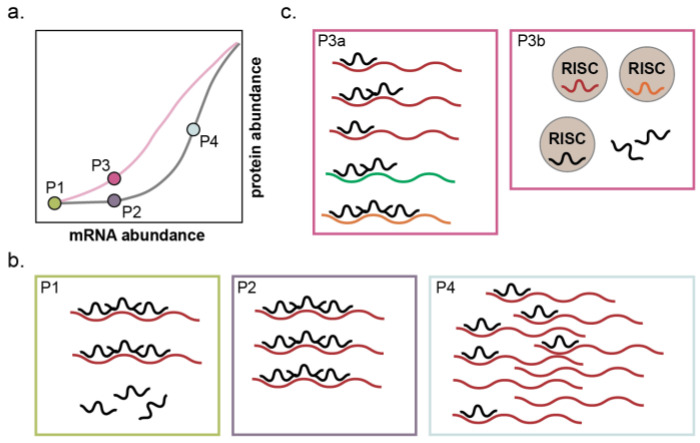
Changes to the miRNA functional unit to miRNA binding site ratio affect protein output. (**a**) A theoretical graph of mRNA abundance to protein abundance for a single target gene as in Figure 2, depicting a high miRNA functional unit to binding site ratio (grey curve) and a lower miRNA functional unit to binding site ratio (pink curve). (**b**) As mRNA abundance of the target gene increases, the initial effect on protein abundance will be minimal (compare P1 to P2). Adequately high mRNA abundance will sufficiently lower the functional unit to binding site ratio as to yield a linear relationship of mRNA abundance to protein abundance and subsequently high protein output (P4). (**c**) If mRNA abundance is static, the functional unit to binding site ratio can still change and yield higher protein output (compare P2 to P3). Depicted examples are the increased expression of mRNAs with competing miRNA binding sites (P3a) or an increase in expression of other miRNAs which compete for incorporation into RISC (P3b).

**Figure 4:**
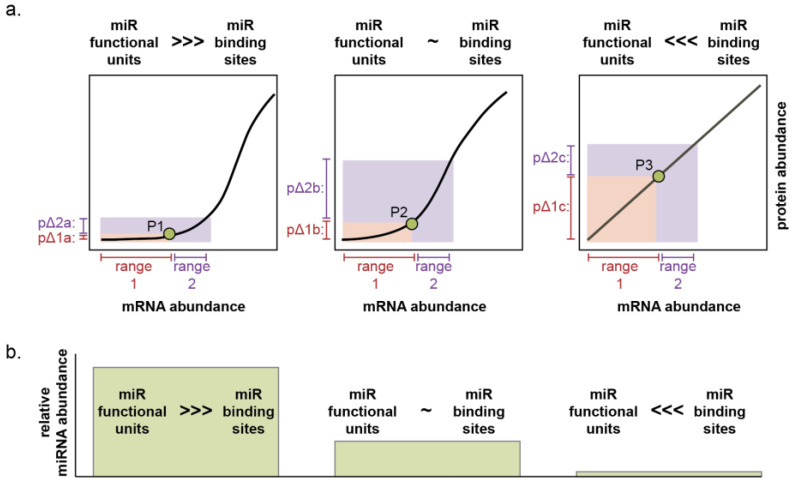
Observed phenotypes are dependent on miRNA functional unit to binding site ratio and target gene abundance. (**a**) The effect of increasing or decreasing mRNA abundance on protein abundance depends on the ratio of miRNA functional units to miRNA binding sites. If functional units are initially in excess, a large range of mRNA abundance is reflected by a comparatively small range of protein abundance (left plot: range 1 and 2 and pΔ1a and pΔ2a). If net functional units are initially comparable to net binding sites, a large range of mRNA abundance still yields minor changes to protein abundance (middle graph: range 1 and pΔ1b), but more significant changes in mRNA will yield observable changes in protein (middle graph: range 2 and pΔ2b). If binding sites are initially in excess, comparable mRNA ranges yield great changes in protein abundance (right graph: pΔ1c and pΔ2c). In all three scenarios, significant over-expression will result in more linear changes in protein abundance. (**b**) Similarly, the effect on protein abundance subsequent to manipulating miRNA levels can be modeled as decreasing from left to right. If miRNA abundance is low, over-expression will cause a profound change in protein output (compare green point P3 to P2). If miRNA is expressed, a similar over-expression will yield minimal impact on protein level (compare P2 to P1). It is noteworthy that the most substantial impact on protein output will occur when the targeting miRNA is initially absent (right graph). This includes the scenarios when the “target” gene is not regulated by the endogenous miRNA at all, but rather is artifactually targeted in the experimental system (such as overexpression of a luciferase gene containing binding sites).

The threshold of mRNA abundance at which this toggle occurs is determined by the stoichiometric ratio between the net abundances of miRNA binding sites and the *accessible* and *functional* miRNA molecules [61,62,63]. This concept of a ratio between net miRNA binding sites and net miRNA functional units is what permits the transcendence of miRNAs from mere inhibitors of gene expression to buffers of cell state. The ratio is a function of the expression of all miRNA target genes; the number of miRNA binding sites on each of those targets; miRNA expression; and incorporation of the miRNA into RISC—this last of which is itself influenced by expression and turnover of the protein components of RISC as well as the expression of other miRNAs competing for incorporation [61,62,63,64] (Figure 3). If a miRNA: target gene network contains significantly more functional miRNA units than miRNA binding sites, the system can absorb large fluctuations in mRNA or miRNA expression with minimal change in protein output (Figure 3, P1 to P2 and Figure 4, left). If functional miRNA units and miRNA binding sites are of approximately equivalent abundance, small fluctuations in mRNA or miRNA expression will result in minimal protein change, but large expression changes in either the miRNA or individual genes within the network can result in substantial influence on protein expression of the network as a whole (Figure 3, P1 to P3 or P1 to P4 and Figure 4, middle). Finally, if functional miRNA units are dwarfed in abundance by miRNA binding sites, small or large changes in the expression of any component can yield proportional changes in protein output (Figure 4, right).

The above framework illustrates how miRNAs establish a molecular regulatory mechanism that is particularly well-suited for defining and stabilizing specific cell types in dynamic systems. The evidence that miRNAs were selected to confer cell state robustness during evolution is compelling. The abundance and diversity of miRNAs in animal genomes correlate with organismal complexity [7,8,65]. Diversification of miRNA expression appears to have evolved in a tissue-specific fashion, and tissue-specific mRNA transcripts have a greater number of miRNA binding sites [13,32,66,67]. During development, these tissue-specific miRNA-targeted networks of transcripts are not well co-expressed with the targeting miRNA within the same cells, but rather, are highly expressed in distinct cell states that are spatially or temporally “adjacent” to the tissue expressing the targeting miRNA—meaning they have very similar, yet still distinct, transcriptomes [14,32]. Such expression patterns suggest miRNAs serve as a buffer to prevent inadvertent differentiation, dedifferentiation, or transdifferentiation “slippage” during development. Experimental evidence is consistent with this model [15,68,69]. For example, during zebrafish development, the presence of miR-430 buffers against both agonists and antagonists of Nodal signaling [68]. Across model organisms, miRNA knockout phenotypes generally occur in stress conditions or with incomplete penetrance [70,71,72,73,74,75]. Taken together, these observations offer compelling evidence that miRNAs evolved to fill a role in bolstering the robustness of developmental programs—maintaining, but not necessarily inducing specific cell states.

### 2.2. Considerations for the Role of miRNAs during Tumor Progression

In order to survive and progress, cancer cells must evolve and, by definition, change. Thus, the observation that miRNAs are globally down-regulated in cancers compared to originating tissue provides further evidence that the primary function of miRNAs is to bolster cell state robustness [66,76]. What concepts drawn from the evolutionary history and developmental roles of miRNAs should be considered when designing and interpreting studies of miRNAs in cancer cells? How might these concepts help explain the discordant literature surrounding miRNAs and tumorigenesis?

**Figure 5:**
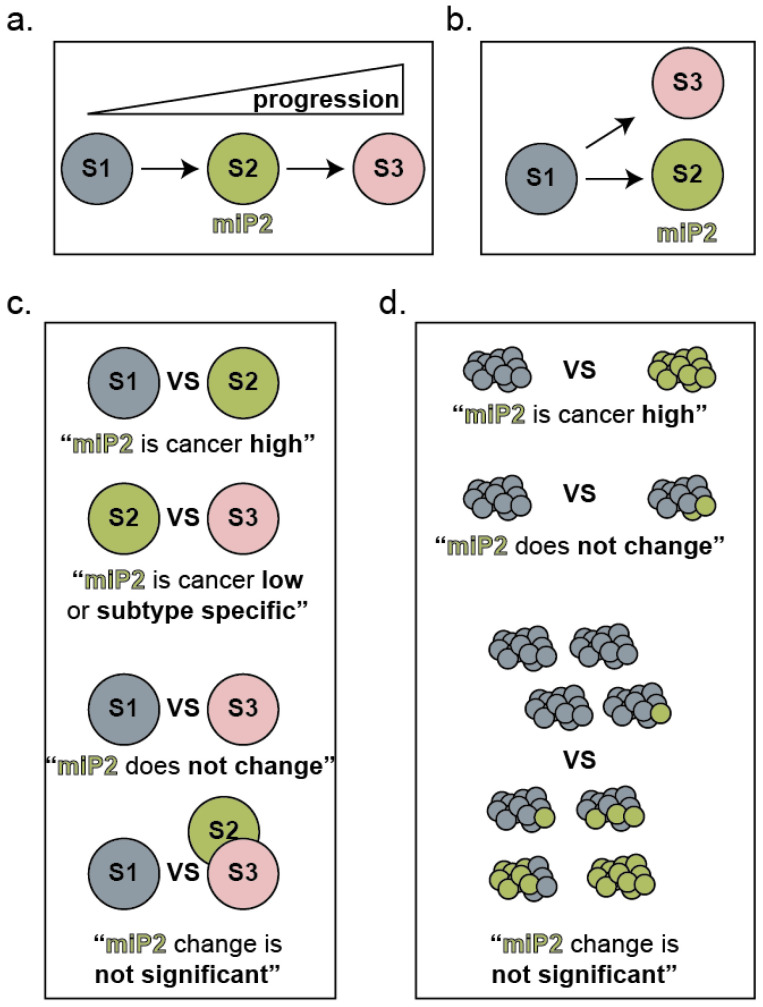
Variations in clinical specimen cohort assembly that can result in inconsistent conclusions. (**a**) A schematic depicting a cancer that has at least three stages of increasing progression (S1, S2, and S3). (**b**) A schematic depicting a cancer with a benign subtype (S1) and two distinct malignant subtypes (S2, S3) that differ in their genetic background (e.g., distinct driver mutations) or environmental context (e.g., distinct sites of distal metastasis). In both schematics, S2 expresses a stage/subtype specific miRNA program (miP2). (**c**) Dependent on the specimens included in each cohort, the observed change in miP2 might be associated with further progression, associated with less progression, associated with a specific subtype, or non-significant. (**d**) Even when cohorts are assembled with consistent staging and subtyping, both inter- and intra-tumoral heterogeneity will influence whether a significant change in miP2 is observed.

The first lesson is the critical importance of the precise cellular ecosystem. That “context matters” is generally well-appreciated among biologists, and the fundamental importance of context when studying miRNA expression and function cannot be over-emphasized. The observed expression levels of a miRNA will depend on the nature and uniformity of the cells harvested, stage in tumor progression, genetic background, and microenvironment (Figure 5). Moreover, calculating the abundance of functional miRNA units theoretically requires the incorporation of the expression of all mRNA targets and all other miRNAs in addition to the specific miRNA of interest. The consequence of manipulating miRNA expression on a target gene is likewise dependent on the baseline expression of all target genes and other miRNAs (Figure 4). For example, over a quarter of melanomas contain genetic amplification of chromosome 1q [77,78]. In a recent study, Xiaonan Xu, Florian Karreth, and colleagues elegantly demonstrate this chromosomal arm is enriched for genes containing repeated miRNA binding sites so that while no single gene alone is sufficiently overexpressed by the genetic amplification of 1q to alter miRNA networks, the culminating consequence on the abundance of competing miRNA binding sites changes the effect of eight miRNAs on cell phenotype [77]. Assessment of copy number variation is not routine in studies utilizing melanoma cell lines. Thus, this critical and common variable affecting whether miRNA manipulation elicits an effect on cancer cell biology would be equally as routinely overlooked.

**Figure 6:**
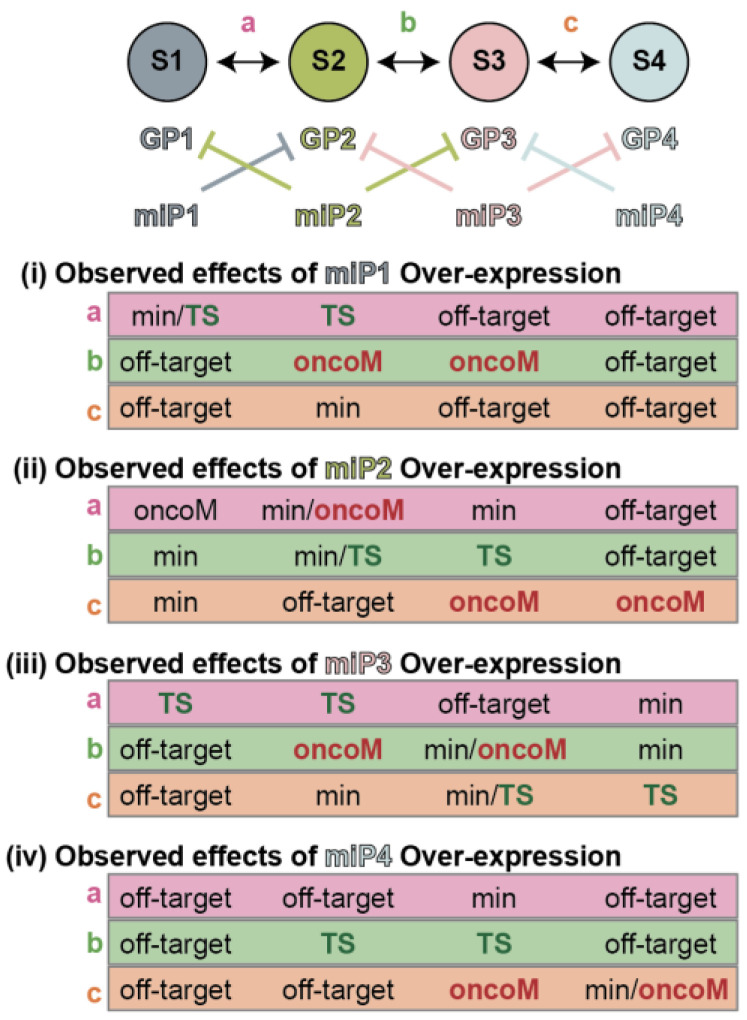
The observed phenotype of miRNA overexpression is a function of cell state and environment. For simplicity, this schematic depicts only four cell states (S1, S2, S3, and S4) and assumes first, that all oncogenic phenotypes increase in severity exclusively from left to right, S1 being the most benign cell state and S4 being the most malignant cell state; and second, that three environmental conditions permit transitions between S1 and S2 (condition “a”), S2 and S3 (condition “b”), and S3 and S4 (conditions “c”). Each cell state contains an associated program of expressed genes (GP1, GP2, GP3, and GP4) and expressed miRNAs (miP1, miP2, miP3, and miP4). Tables (i)–(iv) reveal the expected observed phenotype for overexpression of miRNAs from miP1–miP4, respectively, in each of the four states (S1 to S4, left to right) and each of the four conditions (a to c, top to bottom). Phenotypes are broadly categorized as minimal effect (min), tumor suppressive effect (TS), oncogenic effect (oncoM) or observed effects are likely to be off-target (off-target). Since miRNAs stabilize against change and target programs of expressed genes (GPs) in cell states that are transcriptionally similar, whether the observed phenotype is tumor suppressive or oncogenic depends on whether the GP is expressed in the cell and/or is induced by the environmental condition. For the purposes of this model, if an miP is already expressed, further expression is assumed to have a minimal affect (see Figure 4) and if a targeted GP is not expressed, any observed phenotype is considered to be off-target.

The second lesson is that miRNAs buffer against change caused by internal or external perturbations but may not be the drivers themselves. There are a few notable corollaries to this model. The first is that the phenotypes observed subsequent to miRNA manipulation are dependent on the nature and degree of the internal and external stimuli. For example, miR-430 stabilizes a gene network downstream of Nodal signaling, and thus whether miR-430 appears to be a promoter or inhibitor of Nodal signaling is dependent on whether the system is challenged with an agonist or an antagonist of the pathway [68]. The second is that miRNA expression in tissue does not necessarily report on present cellular phenotypes but rather informs which phenotypes a cell is resistant to acquiring (Figure 6). In the ordered and orchestrated context of development, these two concepts could be argued as one-in-the-same, but in the disordered context of tumor evolution, they cannot be assumed to be equivalent. In the context of cancer diagnosis, it is, therefore, less likely that “tumor-specific miRNAs” will be consistently observed. A consistent tumor-specific miRNA would have to regulate a gene network that was not only anti-tumor but which the cancer cell was also under constant pressure to acquire regardless of the genetic or environmental context. Similarly, the administration of a candidate therapeutic miRNA that targeted a tumor-associated gene network runs the risk of stabilizing that network against further change rather than inhibiting it. If miRNAs serve as buffers against change, then a greater degree of therapeutic efficacy might be expected in contexts where preventing change is advantageous to the patient—chemoprevention, neoadjuvant therapies, or in combination with other therapies to prevent the emergence of resistant populations.

These two lessons suggest that nuances in the assembly of clinical specimen cohorts and study design could substantially change the interpretation of a miRNA’s role in tumorigenesis. Accounting for the precise cell state, tumor stage, and/or uniformity of samples and consideration of the thresholding effect established by the functional miRNA unit: miRNA binding site ratio could aid in clarifying the contributions of specific miRNAs to specific cancers. Melanoma is an example of cancer that presents a high degree of heterogeneity. Nine subtypes of malignant disease are recognized by the World Health Organization, and each of these can arise from a variety of benign precursor lesions totaling over twenty distinct categories of melanocytic neoplasms [79,80]. Layered onto these categories are additional dimensions of heterogeneity, such as the genetic drivers, disease stage, prognostic indicators, and sensitivity to immune checkpoint blockage or targeted therapy. Many studies have profiled the miRNA landscape of melanocytic tumors to identify which molecules are associated with the malignant disease, and functional studies have probed the mechanisms that the miRNAs use to regulate cell behavior. Substantial discordance of observation has been reported. Here we reassess the published literature and available datasets with the conceptional and contextual considerations discussed above.

## 3. MicroRNAs Consistently Associated with Melanoma Progression

### 3.1. Assessment of Melanoma Cohort Assembly in MicroRNA Profiling Studies

We previously conducted a meta-analysis of seven publicly available miRNA profiling studies comparing benign melanoma precursor lesions (melanocytic nevi) to melanomas [47]. We observed that of the 168 miRNAs identified as significantly differentially expressed in at least one study, only seven were reproduced in at least half the studies, and none were reproduced in more than five studies. For the present analyses, we revisited the expanding repertoire of available datasets containing small RNA profiling of melanocytic tumors and considered how nuances of cohort assembly might affect the identification of differentially expressed miRNAs. Based upon the concepts presented in Figure 5 and Figure 6, we hypothesized the following variables could confound observed differences in miRNA expression: gender, age, skin tone, anatomic location of the tumor, sun exposure (chronic and acute) of the lesion, the subtype of the tumor, the genotype of the tumor, the stage of the tumor, tumor cellularity, tumor heterogeneity, tumor immune content and inflammation, comparates, and dermatopathologist team size and concurrence. Gender and age are frequent and well-established sources of heterogeneity in biomarker development [81]. We found that most reports provided these clinical details. Skin tone, anatomic location, and sun exposure are all associated with distinct transcriptional profiles in melanocytes [21]. Likewise, the specific subtype of both benign and malignant tumors, the genotype, and the stage of the disease are associated with different pathologies and/or prognoses, likely indicating diverse transcriptional programs [79]. This information was often, but less frequently, provided by the studies. Tumor cellularity (the portion of a sampled specimen comprising tumor cells), tumor heterogeneity (the portion of a sampled specimen presenting specific stages or histopathological features), immune cell infiltration, and inflammation will all affect the measured transcriptome in sequencing studies and are each rarely provided [47].

“Comparate” refers to specimen type that is considered baseline or normal. In some cases, healthy skin or other types of skin cancers are used as comparates. While this approach is useful for identifying miRNAs that might identify a pigmented skin tumor from other skin, melanocytes constitute less than 5% of the cells in the epidermis, and therefore such comparates will provide limited information on how melanocytic miRNAs change with initiation and progression. In other cases, cultured foreskin melanocytes are used, but such melanocytes are transcriptionally distinct from adult melanocytes, and in vitro media conditions significantly alter miRNA expression [21,82]. Thus, while both approaches yield appropriate comparates for genetic changes acquired during tumorigenesis, both are inappropriate for identifying cell-intrinsic transcriptional correlates of melanocyte transformation.

The dermatopathologist team is an interesting and potentially confounding variable relatively unique to the assembly of cohorts of melanocytic tumors. The assessment of pigmented lesions is a notoriously challenging practice with documented high inter-observer and intra-observer discordance rates [83,84]. Acquiring additional diagnostic opinions from dermatopathologists significantly improves concordance [85]. Studies dissecting the genetic progression of melanoma have used teams of five or more dermatopathologists and documented their consensus (or lack thereof) diagnosis for each specimen to attenuate this confounding factor in cohort assembly [86]. Few miRNA profiling studies provide evidence in methods, authors, and contribution sections for the use of dermatopathology teams in cohort assembly.

Ultimately, we restricted our further analyses to studies that clearly identified the disease subtypes in each cohort and attributed at least one dermatopathologist was attributed as reviewing each specimen. We identified twenty-one studies that met these criteria [47,87,88,89,90,91,92,93,94,95,96,97,98,99,100,101,102,103,104,105,106].

### 3.2. Cross-Study Concordance of MicroRNAs Associated with Melanocytic Nevi

We first assessed the concordance of individual miRNA differential expression when restricting comparisons to melanocytic nevus versus primary melanomas (9 comparisons); nevus versus melanoma metastasis (6 comparisons); and primary melanomas versus metastasis (10 comparisons). For each comparison, each miRNA was assigned a value based on the number of studies in which it was identified as differentially expressed. Of the 468 statistically significant associations reported across studies, 365 (78%) were not reproduced in any other study, and 59 (16%) were observed in three or more studies. We reasoned that were stage-specific miRNAs to exist (as depicted in Figure 6), they should consistently present as enriched in cohorts of that stage, regardless of which other stages are used for comparison. For example, nevus-specific miRNAs would present as enriched to nevus cohorts whether compared to primary melanomas or melanoma metastasis cohorts; primary melanoma-specific miRNAs would present as enriched to primary melanoma cohorts whether compared to melanocytic nevi or melanoma metastasis cohorts, and so forth. We identified only three miRNAs that met these criteria: miR-211, miR-204, and miR-21 (Table 1). All three were identified as specific to the nevus cohorts, with miR-211 and miR-204 (both miRNAs from the same family) being enriched in nevi and miR-21 being depleted in nevi.

Another possible pattern is a miRNA that is continuously and incrementally down-regulated at each stage of progression. We would expect such a miRNA to exhibit enrichment in the nevus cohorts when compared to the primary melanoma cohorts; enrichment in the primary melanoma cohorts when compared to the melanoma metastasis cohorts; and an even more pronounced or consistent enrichment in the nevus cohorts when compared to the melanoma metastasis cohorts. The inverse would be true for a miRNA that is continuously and incrementally up-regulated at each stage of progression. No miRNAs presented this precise pattern, although two patterns were suggestive—those of miR-200B (down-regulated with progression) and miR-142 (up-regulated with expression). However, in both cases, the miRNAs were differentially expressed with the least consistency when the melanocytic nevus cohorts were compared to melanoma metastasis. As discussed in the next section, one explanation for this observation is the miRNAs are indeed increasingly dysregulated with each stage of progression but that the melanoma metastasis cohorts comprise more heterogeneous specimens.

### 3.3. Increased Variability of miRNA Expression Is Associated with Melanoma Progression

Two reoccurring patterns in our analysis suggested that the cohorts of melanoma metastasis, and to a lesser degree, primary melanomas, contained greater heterogeneity of specimens than the melanocytic nevus cohorts. First, we observed generally less concordance for any comparison that included the melanoma metastasis cohorts. This includes comparisons of nevus cohorts to melanoma metastasis cohorts where, at the extreme ends of progression, we intuitively expected to observe the most significant differences in expression. Second, we did not observe any miRNAs that presented the patterns for stage-specific expression expected for the melanoma cohorts (Table 1). To reiterate, we expect a stage-specific miRNA to be consistently enriched in at least three cohorts compared to either of the other stages (an arguably low bar of reproducibility when twenty-one studies are being considered). Nine miRNAs were consistently enriched in primary melanoma when compared to melanoma metastasis but not when compared to nevi. At first consideration, this pattern could be indicative of miRNAs that are expressed in cutaneous tumors that are then lost upon metastasis. However, if true, we would expect each to be enriched in nevus cohorts when compared to melanoma metastasis—a pattern that is not observed. Similarly, we observed eleven miRNAs that were consistently enriched in nevus cohorts compared to primary melanoma cohorts but not when compared to melanoma metastasis cohorts.

Taken at face value, these two patterns could suggest that most miRNAs that change in expression upon transformation subsequently revert to their original expression upon metastasis. We favor an alternative explanation—that the melanoma metastasis cohorts are substantially more heterogeneous than either the nevus or the primary melanoma cohorts. Heterogeneity would result in a greater variance of miRNA expression across each cohort and, consequently, reduce the probability of identification as “significantly differentially expressed.” Such heterogeneity could come from a variety of sources. It is possible that intertumoral heterogeneity is increased across metastatic tumors, such that there are effectively different subtypes expressing distinct miRNAs (Figure 5b). It is possible that intratumoral heterogeneity is increased in metastatic tumors (Figure 5c,d). Since, by definition, all nevus and primary melanomas considered here are cutaneous and melanoma metastasis can occur at a variety of anatomic locations, it is possible that the non-tumor cell component of each specimen adds greater heterogeneity to these latter cohorts, reducing the potential for observed significant differential expression (Figure 5d).

We considered another possible explanation for the lack of reproducible melanoma metastasis-specific miRNAs. Whereas miRNAs presumably evolved to aid in the ordered processes of development, tumorigenesis presents a higher degree of disorganization. In melanoma, although about 45% of the changes in the transcriptome do appear as sequential dedifferentiation or “development, but in reverse”, the other 55% display no such pattern [21]. MiRNAs are organized into families dependent on sequence similarities and, presumably, consequent similarities in target networks [107]. In the context of development, specific promoters drive specific miRNA family members in concordance with a developmental program. However, in the case of tumorigenesis, it is possible that any aberrantly expressed loci that stabilize an advantageous behavior for the evolving cancer cell will be selected. Indeed, the mutually exclusive expression of either MIR211 or MIR204 family members in melanoma has been elegantly demonstrated by several groups [108,109]. We reasoned that if we considered full miRNA families, a greater degree of concordance might emerge from the compared cohorts. Five miRNA families emerged as consistently enriched in nevus cohorts compared to primary melanomas, including MIR204/211, MIR23, MIR141/200, MIR203, and MIR10/100 (the last of which contains the MIR125 miRNAs) (Table 2). The MIR200 and MIR125 miRNAs frequently appear in the melanoma literature, and this analysis would suggest the expression and activity of each family should be considered collectively. Two families—MIR204/211 and MIR141/200—were enriched in at least three comparisons of nevus to melanoma metastasis (Table 2). Notably, across all families, the coefficient of variance was higher when comparing nevus cohorts to melanoma metastasis than when comparing nevus cohorts to primary melanomas. This observation is consistent with the interpretation that the melanoma metastasis cohorts harbor greater heterogeneity than the primary melanoma cohorts.

## 4. miR-211-5p Expression Confers Robustness to Pigmented Cells

### 4.1. miR-211-5p Is a Consistent Nevus-Associated miRNA

We next sought to assess the literature exploring the biological role of miRNAs in melanoma progression, with a focus on those most consistently dysregulated in profiling studies, specifically, the MIR211/MIR204 family. We recently compared the full transcriptomes of melanocytic nevi to adjacent melanomas arising from each nevus and observed miR-211-5p as the single most consistent and highly expressed nevus-enriched transcript, an observation that corroborated prior fluorescent in situ hybridization studies [47,82,110,111]. These data support the hypothesis that miR-211-5p, in particular, is crucial for establishing and stabilizing transcriptional programs of nevus melanocytes. In human anatomy, miR-211-5p is largely restricted to the melanocytic lineage, with notable expression in the skin and the retina, which are both pigmented tissues [112]. Presumably, the miR-211-5p regulated gene networks that emerged through evolutionary pressures are expressed in cell types that are “transcriptionally adjacent” to the cell state of pigment-producing melanocytes. These cells might include melanocytes at various degrees of pigmentation (sun-exposed versus not [113]); precursors to melanocytes in developing or adult skin (melanoblasts [114], follicular stem cells [115], healing tissue [116], adult epidermal melanocyte stem cells [21,117]); or different types of adult melanocytes (amelanotic v-mels [21]). Although these “melanocyte adjacent” cell states might contain the evolved target networks of miR-211-5p, expression of the miRNA is also dysregulated in several cancers of non-melanocytic origin, including ovarian cancer, breast cancer, glioblastoma, and others [53]. Unless the miRNA is part of an evolved and organized response to suppress tumor progression in each of these tissues, the genes regulated by aberrant miR-211-5p expression in these contexts are more likely to be “off-target”—meaning the consequence of coincidental targeting, as opposed to a coordinated gene network selected through adaptation.

The effect of miR-211-5p on target genes and cell behavior in both melanocytic and non-melanocytic contexts has been recently and well-reviewed [53,54]. In these various contexts, miR-211-5p has been incongruously identified as a potent tumor suppressor [104,108,118,119,120,121], a potent oncogene [108,109,112,122,123,124], a critical mediator of therapeutic resistance [109]; a biomarker of better overall survival [125], a biomarker of worse overall survival [124], and an excreted extracellular signaling molecule capable of reprogramming distal cells [124,126]. We have assessed whether the observed effect of miR-211-5p on tumorigenesis can be harmonized through consideration of the precise context of miR-211-5p expression. Specifically, we analyzed experimental systems that model the clinical circumstances under which miR-211-5p expression is most consistently altered—the transformation of a melanocytic nevus to a melanoma. We considered how cellular, environmental, and genetic contexts could influence disparate observations. Below we discuss a variety of studies and experiments that use different techniques to measure or manipulate the gene (“mir-211”), primary transcript (“pri-mir-211”), precursor transcript (“pre-mir-211”), mature transcript (“miR-211-5p”), synthesized mimics (“shmiR-211; miR-211-5p”), and inhibitors (“anti-miR-211; miR-211 inhibitor; miR-211 sponge”). For simplicity we will adopt the generic nomenclature “miR-211” to describe expected net mature miR-211-5p functional units.

### 4.2. Experimental Conditions Influence Observed miR-211 Expression and Function

Cultured primary normal human epidermal melanocytes (NHEM) are reported to express more miR-211 as compared to cultured human melanoma cell lines, suggesting these in vitro systems successfully model this molecular characteristic of nevus transformation [112,118,119,127,128,129]. However, in addition to directionality, the magnitude of differential expression should also be considered when assessing the biological function of a miRNA (Figure 2, Figure 3 and Figure 4). MiR-211 is usually detectable in primary melanoma specimens, and its expression is approximately 2–10-fold greater in melanocytic nevi [47,87,88,89,90,91,92,93,94,95,96,97,98,99,100,101,102,103,104,105,106]. In comparison, expression of the miRNA is routinely reported at 10^2^–10^4^-fold greater in cultured NHEMs, as compared to cultured human melanoma lines [112,118,127,129]. Just as frequently, a greater than 10^8^-fold decrease or no detection at all is reported in cultured human melanoma lines [112,118,119,127]. Other comparisons of NHEMs and melanoma lines contradict these observations, reporting only minor (2-fold) or no change of expression of miR-211 between NHEM and melanoma cultures [112,128,130]. We wondered whether the reported expression discrepancies across in vitro studies and the stark differences in magnitude between in vitro and ex vivo specimens could be the consequence of experimental context.

Cultured melanoma cell lines can be broadly categorized as either “established”—meaning they are of significantly high (often unknown) passage since derivation and have reached a large degree of uniformity in culture—or short-term “primary” cultures—meaning they are plated ex vivo and retain more characteristics and heterogeneity of the original tumor [131]. Established cell lines undergo genetic and transcriptomic drift away from the primary tumor during culture [132]. A recent elegant study demonstrated that in primary melanoma cultures, this phenotype drift is drastically accelerated in culture media containing high concentrations of tyrosine [133]. Not only was phenotype drift accelerated, it was also directional—inducing a non-pigmented phenotype within eight passages. Among the most down-regulated genes in the high tyrosine media were *MITF* and *TRPM1* (each by approximately 10^3^-fold). *MITF* directly activates the shared promoter of *TRPM1* and miR-211, and, therefore, both pigmentation and the expression of *MITF* and *TRPM1* are well-correlated surrogates for miR-211 expression [108,112,128,129,134]. These data suggest that established melanoma cell lines (greater than eight passages) grown in high tyrosine media (including base media DMEM and RPMI) have drifted toward miR-211 expression that is upwards of a thousand-fold less than in the original clinical specimen. Similarly, NHEM media conditions influence the transcriptome. Switching between two commonly used NHEM media—PMA-free and PMA-containing—rapidly induces a reversible 6–10-fold increase in miR-211 expression [82,112]. Taken together, we conclude the choice of media for NHEM and melanoma cultures could result in the net gain or loss of 10^4^ relative miR-211 expression—a substantial difference in comparison to the less than 10-fold change observed in nevus and primary melanoma clinical specimens. Supporting this interpretation, reported observations of 10^2^ or greater differences in miR-211 expression between cultured NHEMs and melanoma cells universally came from experimental conditions using PMA-containing and high tyrosine media, respectfully [118,127,129], whereas experiments that used PMA-free and/or low tyrosine media presented 2-fold or less differential expression [128,130].

**Figure 7:**
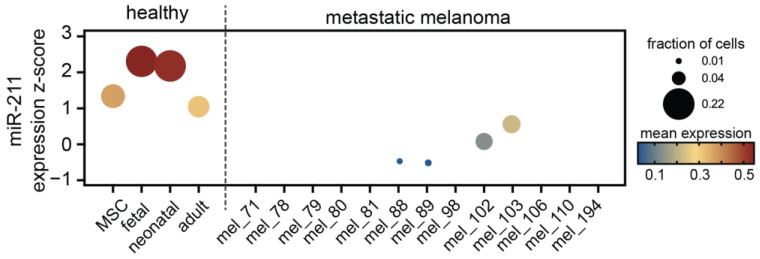
The expression of miR-211 in human epidermal melanocytes in skin. The expression of miR-211 in published single cell RNA-sequencing datasets from either fresh human epidermal melanocytes (Belote, et al. (2022) [21]) or fresh human melanoma metastasis (Tirosh, et al. (2016) [136]) is displayed. Data are presented as the fraction of cells with miR-211 read-counts (circle size); mean expression (circle color) and z-score compared to other cell types (y-axis). MSC, melanocyte stem cell.

It is important to note that it remains unclear which, if any, of the NHEM culture conditions faithfully recapitulates the expression of miR-211 in human skin. Since the presence of PMA induces greater melanocyte pigmentation, it has been suggested cells in these conditions are more differentiated, whereas cells in PMA-free media are more similar to melanocyte precursors termed “melanoblasts” [135]. Recently, we developed a human melanocyte atlas by conducting single-cell RNA sequencing of fresh ex vivo epidermal melanocytes through development and in adult skin [21]. The expression of miR-211 is indeed greater in neonatal foreskin as compared to melanocyte precursors (MSC), but then drops substantially in adult epidermal melanocytes (Figure 7).

We are not aware of a dataset that directly compares miR-211 expression in adult epidermal human melanocytes in situ or ex vivo to cultured NHEMs in different media conditions. We, therefore, cannot comment on which culture conditions promote miR-211 expression that is most similar to the in situ cells, but if miR-211 does function to establish thresholds for cell behaviors, changes in the baseline and relative expression by 3–4 orders of magnitude could substantially alter experimental outcomes (Figure 4). For example, in one study that assessed the effect of miR-211 expression on tumor growth, a change in expression of 600-fold in a melanoma cell line that expresses negligible baseline miR-211 resulted in a 20-fold change in phenotype (tumor volume and weight), whereas in two lines expressing higher baseline miR-211, both 25- and 25,000-fold increases in expression resulted in only a 1.5–2-fold change in phenotype [122]. Similar examples, where the magnitude of a phenotype change was more dependent on the baseline miR-211 expression than the level of additional induced expression, are in the literature [108,109,120].

**Figure 8:**
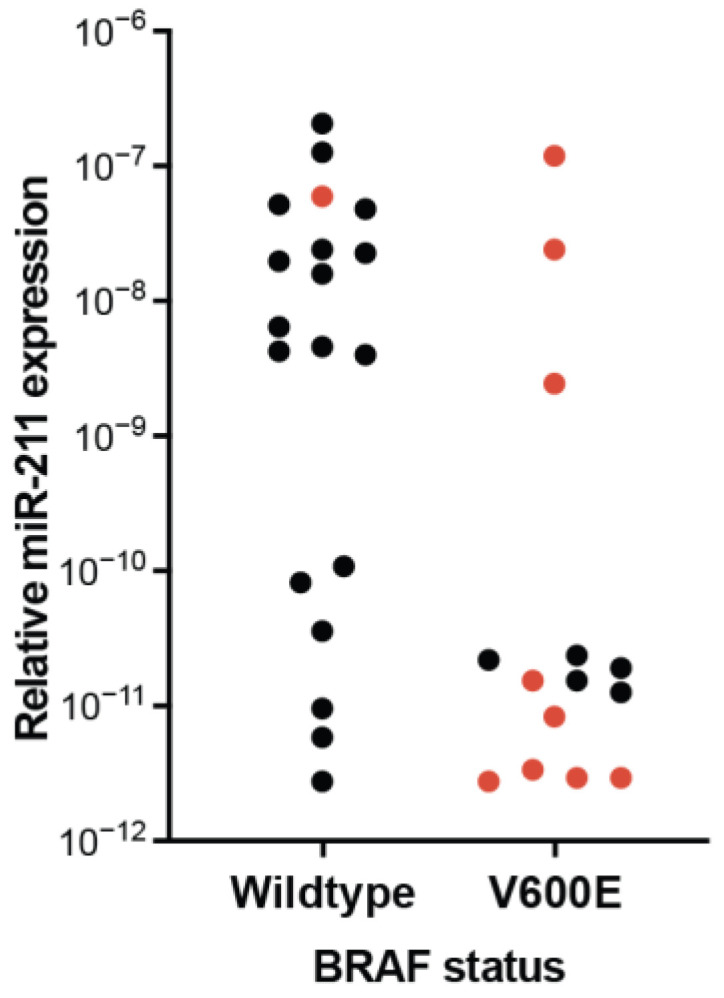
BRAF status is related to miR-211 expression. Relative miR-211 expression from either *BRAF* wildtype or *BRAF^V600E^* cell lines. Color indicates either resistance (black) or sensitivity (red) to targeted *BRAF^V600E^* inhibitor. Reanalysis of supplementary data presented in Lee, et al. (2021) [122].

We identified other experimental conditions that could be contributing to discrepancies in reported phenotypes. One is the genetic landscape of the cells used. Cutaneous melanomas can be broadly categorized into major subtypes by driver mutation: *BRAF* activating, *RAS* activating, *NF1* loss (sometimes grouped with *SPRED1* loss), and triple wild type (sometime *KIT* mutant is separated from this group) [80,137]. *BRAF^V600E^* is the most common driver of both melanoma and melanocytic nevi [138,139]. Genetic alterations that commonly occur during the transformation of a nevus to a melanoma include deep loss of *CDKN2A* (~60% of cases), *TP53* alteration, and *PTEN* loss (each approximately 20% of cases) [110]. We found multiple instances in the literature where the genotype of cultured cells appeared to affect observed miR-211 expression and associated phenotypes. In a pioneering study, Boyle and colleagues reported that miR-211 manipulation affected cellular invasion, but only in some melanoma cultures [112]. The authors not only stratified the affected and non-affected cell lines by baseline MITF and miR-211 expression, but also by genotype. All of the MITF-low lines harbored deep loss of *CDKN2A* whereas all MITF-high lines were wildtype at this locus, which suggests that tumor suppressor loss might alter baseline miR-211 expression and observed phenotype. Elevated miR-211 expression has also been associated with innate resistance to therapies specifically targeting *BRAF^V600E^* [122,123]. However, there is an additional association between wildtype *BRAF* and increased miR-211 expression in melanoma cell lines, indicating the inverse correlation between miR-211 expression and therapeutic sensitivity might be partially explained by the poor expression of miR-211 in cell lines harboring the therapeutic target (BRAF^V600E^) (Figure 8). Finally, we recently demonstrated that the effect of media conditions that induce miR-211 expression on NHEMs was dependent on *BRAF* status as well. In the presence of *BRAF^V600E^*, PMA-containing media induces growth arrest, whereas, in the absence of the oncogene, it supports proliferation [82].

**Figure 9:**
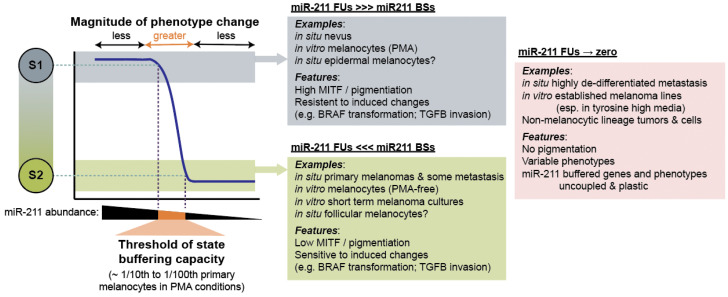
miR-211 establishes a threshold effect on phenotype switching. This schematic summarizes our conclusions from the literature reanalysis. Melanocytes can occupy at least two distinct states (S1 and S2). At higher expression levels, miR-211 functional units (FU) are much greater than miR-211 binding sites (BS), thus establishing a robust S1 gene network (grey boxes), such that changes in miR-211 expression result in minimal alternations to phenotype. At levels below a threshold (benchmarked at 1/10th to 1/100th the level in primary neonatal melanocytes grown in PMA-containing media), miR-211 FUs are much less than miR-211 BSs and the melanocytes are sensitive to induced change into S2 (green boxes). Both scenarios are distinct from contexts where miR-211 FUs are approaching zero (pink box). Examples and features of each context are summarized.

Another experimental condition that could be contributing to discrepant results is the source of both NHEM and melanoma cell cultures. Most studies utilize NHEMs derived from neonatal foreskins as opposed to adult skin. The transcriptional programs of neonatal epidermal melanocytes and adult epidermal melanocytes in situ are distinct, including a marked difference in the MITF program [21] and miR-211 (Figure 7). Likewise, in studies that use adult NHEMs as comparates in culture, the observed difference in miR-211 expression compared to melanoma cultures was abrogated [128]. Melanoma cultures are derived from either primary or metastatic tumors. The expression of miR-211 appears to be more variable in cohorts of metastatic specimens as compared to primary tumors (Table 1 and Table 2). Our reanalysis of single-cell RNA sequencing from fresh metastatic melanomas revealed substantial intra- and intertumoral heterogeneity of miR-211 expression (Figure 7) [136]. In the literature, many lines derived from melanoma metastasis frequently presented no detectable levels of miR-211. Based upon the theory of miRNA discussed above, we would expect a fundamental difference in phenotype between a “low” miR-211 context (the target network is established, and the threshold for phenotype-switching is lower) versus a “no” miR-211 context (the target network, and therefore threshold effect, is not established). For example, when miR-211 is constitutively over-expressed, melanoma cells in xenograft models grow more rapidly in the primary location but lose the ability to metastasize [140]. When a miR-211 inhibitor is constitutively over-expressed, melanoma cells in xenograft models exhibit, again, accelerated tumor growth, but now coupled to increased metastasis [141]. In contrast, when miR-211 is knocked out entirely, implanted melanoma cell exhibit significantly reduced growth [123]. If miR-211 is considered an inhibitor of genes, these three sets of observations appear contradictory. As an alternative interpretation, the experiments are consistent with the model that miR-211 establishes a threshold for phenotype switching from a proliferative to an invasive state. The threshold can be increased or decreased, altering the cellular response to induced phenotype changes but it is critically important for these cells that the threshold is established in order to stabilize either phenotype (Figure 9).

### 4.3. MicroRNA-211 Establishes Thresholds for Target Gene Expression and Cell Phenotypes

**Figure 10:**
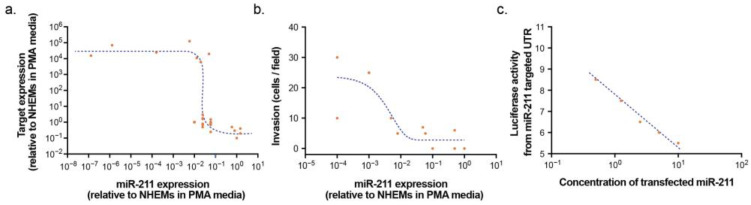
Evidence of miR-211 target gene and phenotype threshold effect. (**a**) Relative expression of miR-211 (compared to NHEMs in PMA-containing media) plotted against relative expression of validated targets (measured by RT-qPCR). (**b**) Relative expression of miR-211 plotted against transwell invasion efficiency. (**c**) Co-transfection of synthesized mature mimic and luciferase reporter construct containing miR-211 target sites. Concentration of transfected miR-211 synthesized mature mimic plotted against arbitrary luciferase units. Data are estimated and reanalyzed from graphs published in Levy, et al. (2010) [129]; Sakurai, et al. (2011) [127]; Bell, et al. (2014) [120]; Díaz-Martinez, et al. (2018) [109]; and Lee. et al. (2021) [122].

The above interpretations suggest that miR-211 establishes thresholds for phenotype switching in melanocytic cells. We, therefore, explored the literature for evidence that would support or refute the role of miR-211 in establishing thresholds in target gene expression and discrete cellular phenotypes. Using the relative expression of neonatal NHEMs in PMA-containing media and specific melanoma cell lines (A375 and SK-MEL-28) in high tyrosine media as benchmarks common to multiple studies, we assessed the effect of disparate miR-211 levels on validated target mRNA (*NUAK1/ARK5*, *IGFBP5*, *PRAME*, *TGF-βRII*, *SLUG*, and *CHD5*) measured by RT-qPCR [109,122,127]. We observed a relationship that is supportive of a threshold effect (Figure 10a). Changes in miR-211 across 10^−1^–10^−2^ relative to NHEMs in PMA-containing media were associated with large changes in target gene expression, whereas altering miR-211 expression above or below this window had minimal effect.

In order to assess the relationship between miR-211 and cell phenotype, we utilized data from Levy and colleagues’ seminal report that established intronic miR-211 as the tumor suppressor encoded with the host *TRPM1* gene [129]. We plotted relative miR-211 expression against the absolute metric of cellular invasion across a basement membrane (cells/field in a transwell assay). Similar to target gene expression, we observed evidence for a phenotype threshold effect (Figure 10b). At miR-211 expression levels above 10^−1^ compared to NHEMs in PMA-containing media, induced invasion was minimal and not correlated with miR-211 expression. Below this miR-211 expression threshold, the invasion was more readily induced in a dose-dependent manner. In contrast, when a luciferase reporter with a miR-211 target sequence and miR-211 were both simultaneously over-expressed in non-melanocytic cells lacking miR-211 or target network expression, a linear dose-responsive relationship was observed (Figure 10c), indicative of a classic direct inhibition curve as expected in this context (Figure 4, right) [120].

## 5. Perspectives, Future Directions, and Conclusions

Our intent in this review was to reanalyze the existing and often discordant literature on miRNA expression and function in melanoma and determine whether models derived from the fundamental theory of miRNAs as establishers of cell state robustness would permit more concordant interpretations. We first analyzed the cohorts of clinical specimens assembled for miRNA profiling studies. We determined that of the potential sources of confounding covariates, few were routinely considered or recorded. By considering only cohorts with consistent subtyping, subclassifying melanomas into primary and metastatic groups, consolidating miRNAs into functional families, and monitoring for consistent patterns of concordance, two families—MIR211/204, MIR141/200—emerged as enriched in the nevus state and one miRNA—miR-21—as depleted in the nevus state. However, no state-specific miRNAs for melanoma metastasis were observed, which, we speculate, is due to heterogeneity that is either inherent to the biology of metastatic disease or instilled in these cohorts by confounding covariates. We previously demonstrated that one method to combat heterogeneity in existing cohorts of nevi and primary melanomas is to consider ratios of the top differentially expressed miRNAs [47]. Whether the success of this method is due to an alternative form of normalization that effectively controls for tumor cellularity or because it more accurately measures miRNA functional units by accounting for other highly expressed miRNAs (Figure 3c) remains to be established. Such an approach might aid in the identification of consistent metastasis-associated miRNAs but would first require the assembly of a training cohort with more refined subclassing (genetic driver, immune component, tumor size, tumor site, transcriptomic classification, etc.).

It is also a possibility that due to the inherent disorganization of tumorigenesis, no reliable miRNA marker of melanoma metastasis can be identified. In this case, the detection and quantification of miRNAs that stabilize the more homogenous benign state would yield more informative biomarkers. The MIR211/MIR204 family was one such consistent identifier of the benign nevus cell state. However, it is important to note that existing profiling studies of miRNA expression focus almost exclusively on the most common forms of melanocytic nevi and cutaneous melanoma. A fluorescent in situ hybridization study found that while miR-211 was also consistent as a marker of Spitzoid nevi, the miRNA was not down-regulated in Spitzoid melanomas [111]. Thus, determining the expression pattern of this miRNA in other subtypes of melanocytic tumors will be crucial for further biomarker development.

We analyzed in vitro culture systems that attempt to recapitulate the approximate cell type, genotype, and miR-211-5p expression levels of the in situ tissue and disease. In these systems, we present evidence for thresholds of both gene expression and cellular phenotype established by miR-211-5p. The threshold limit appears to be approximately 10–100-fold below that of human neonatal melanocytes grown in pigmentation-inducing media, comparable to the 2–10-fold down-regulation observed in primary melanomas. When expressed above this threshold, the buffering effect of miR-211-5p against oncogene-induced proliferation and invasion is remarkably consistent and congruent with clinical specimen profiling [82,104,108,112,118,119,120,121,140]. The expression of miR-211-5p in metastatic melanomas and derived melanoma lines is more variable and often approaches zero. The environmental context, immune component, and genome of metastatic tumors are more heterogenous than that of melanocytic nevi and primary melanomas in the skin. Thus, while the reported phenotypic effects of miR-211-5p in these systems are more variable, we expect this could be due to what is ultimately a set of experimental parameters that are more challenging to define.

Overall, our analyses strongly support the role of miR-211-5p as a buffer against melanocytic cell state transitions, specifically able to stabilize against the acquisition of invasive or oncogene-induced proliferative phenotypes. These observations provide evidence for the theory that the biological function of miRNAs is to confer robustness to specific cell states.

## Figures and Tables

**Table 1:** Concordance of differentially expressed miRNAs across studies. If a miRNA was identified as differentially expressed it was assigned a value of 1 if enriched or -1 if depleted per study. Values were summed across studies (*n* = 9 for column 1, *n* = 10 for column 2 and *n* = 6 for column 3). Only those associations with net values of 3 or more are shown. MiRNAs were considered stage-specific if they appeared as stage enriched in both comparisons (i.e., the expected pattern for nevus-specific miRNAs would be green in columns 1 and 3; for metastasis-specific miRNAs would be red in columns 2 and 3; and for primary melanoma-specific miRNAs would be red in column 1 and green in column 2). Green cells indicate positive association and red cells indicate negative association. MN, melanocytic nevi; PM, primary melanoma; MM, metastatic melanoma.

miRNA	Nevus to Primary Melanoma	Primary Melanoma to Melanoma Metastasis	Nevus to Melanoma Metastasis
**miR-23B**	**6**		
**miR-125B**	**6**		
**miR-211**	**5**		**3**
**miR-204**	**5**		**3**
**miR-125A**	**4**		
**miR-455**	**4**		
**LET7-A**	**4**		
**LET7-B**	**3**		
**miR-100**	**3**		
**miR-183**	**3**		
**miR-149**	**3**		
**miR-99A**	**3**		
**miR-26B**	**3**		
**miR-155**	**-4**		
**miR-21**	**-5**		**-3**
**miR-205**	**5**	**3**	
**miR-141**	**4**	**5**	
**miR-200A**	**4**	**4**	
**miR-203**	**4**	**4**	
**miR-200B**	**3**	**5**	**3**
**miR-200C**		**4**	
**miR-142**	**-4**	**-3**	**-3**
**miR-224**		**4**	
**miR-203A**		**4**	
**miR-29C**		**-3**	
**miR-218-2**		**-3**	
**miR-326**		**-3**	
**miR-4491**		**-3**	
**miR-625**		**-3**	
**miR-675**		**-3**	
**miR-766**		**-3**	
**miR-215**		**-4**	
**miR-3130**		**-4**	
**MN-specific**	**5**		**5**
**PM-specific**	**-5**	**5**	
**MM-specific**		**-5**	**-5**

**Table 2:** Concordance of differentially expressed miRNA families across studies. MicroRNA families were identified from miRbase.org. For each study, an expression value was assigned by dividing the number of family members identified as differentially expressed by the total number of family members. CV, coefficient of variance. Studies included were Torres, et al. (2019) [47], Kozubek, et al. (2013) [90], Liu, et al. (2012) [92], Xu, et al. (2020) [105], Wandler, et al. (2017) [100], Xu, et al. (2012) [104], Sand, et al. (2013) [96], Komina, et al. (2016) [89], Schulz, et al. (2008) [97], Chen, et al. (2010) [87], Segura, et al. (2010) [98] and Lu, et al. (2019) [94].

Family	Nevus to Primary Melanoma									CV	Nevus to Melanoma Metastasis					CV
**MIR204/211**	1	1	0.5	0.5	1	0	0.5	0.5	0	**0.7**	1	0.5	0.5	0	1	**1.04**
**MIR23**	0.5	0.5	0	0.5	1	0	0.5	0	0.5	**0.86**	0.5	0	0	0	0	**2.24**
**MIR141/200**	0	0	0.8	0	0.4	0.6	0.4	0	0.4	**1.04**	0.6	0.8	0.6	0	0	**1.2**
**MIR203**	0	0.5	0.5	0.5	0.5	0.5	0	0	0	**0.95**	0.5	0.5	0	0	0	**1.37**
**MIR10/100**	0.38	0	0.38	0.25	0.5	0.13	0.13	0	0.25	**0.78**	0.25	0.38	0	-0.13	0	**1.68**
	**[47]**	**[90]**	**[92]**	**[105]**	**[100]**	**[104]**	**[96]**	**[89]**	**[97]**		**[87]**	**[92]**	**[96]**	**[98]**	**[94]**	

## Data Availability

Single cell RNA-seq datasets were obtained from publicly accessible repositories: GSE151091 and GSE72056.
